# Comparative evaluation of 0.2% Chlorhexidine Mouthwash, Xylitol Chewing Gum, and Combination of 0.2% Chlorhexidine Mouthwash and Xylitol Chewing Gum on Salivary *Streptococcus mutans* and Biofilm Levels in 8- to 12-Year-Old Children

**DOI:** 10.5005/jp-journals-10005-1384

**Published:** 2016-12-05

**Authors:** Meena Syed, Radhika Chopra, Vandana Shrivastava, Vinod Sachdev

**Affiliations:** 1 Postgraduate Student, Department of Pedodontics and Preventive Dentistry, ITS Centre for Dental Studies and Research, Ghaziabad, Uttar Pradesh, India; 2 Reader, Department of Pedodontics, ITS Centre for Dental Studies and Research, Ghaziabad, Uttar Pradesh, India; 3 Associate Professor, Department of Microbiology, ITS Centre for Dental Studies and Research, Ghaziabad, Uttar Pradesh, India; 4 Professor,Department of Pedodontics, ITS Centre for Dental Studies and Research, Ghaziabad, Uttar Pradesh, India

**Keywords:** Biofilm, Chlorhexidine, *Streptococcus mutans*, Xylitol.

## Abstract

**Aim:**

To assess the effect of combining 0.2% chlorhexidine (CHX) mouthwash with xylitol (XYL) chewing gum on *Streptococcus mutans* and biofilm levels among 8- to 12-year-old children.

**Materials and methods:**

Sixty children aged 8 to 12 years were selected with moderate and high salivary S. *mutans* levels. They were divided into three groups of 20 children each: (1) XYL group where the subjects chewed XYL twice daily; (2) CHX where rinsing was done twice daily; and (3) combination of XYL and CHX group (XYL+CHX) where both the agents were used once daily. The *S. mutans* colony-forming units (CFUs) were counted by using the mitis salivarius agar plate at the beginning of the study and at 15 days, 1, 2, and 6 months from the start of the study.

**Results:**

The XYL+CHX group showed the maximum reduction in both the biofilm and S. *mutans* scores throughout the study period.

**Conclusion:**

The XYL+CHX combination reduced both the biofilm and S. *mutans* score significantly better than either XYL chewing gums or CHX mouthwash used alone.

**How to cite this article:**

Syed M, Chopra R, Shrivastava V, Sachdev V. Comparative evaluation of 0.2% Chlorhexidine Mouthwash, Xylitol Chewing Gum, and Combination of 0.2% Chlorhexidine Mouthwash and Xylitol Chewing Gum on Salivary *Streptococcus mutans* and Biofilm Levels in 8- to 12-Year-Old Children. Int J Clin Pediatr Dent 2016;9(4):313-319.

## INTRODUCTION

Dental caries is an infectious disease commonly found in the oral cavity. W.D. Miller had postulated the chemo-parasitic theory for the formation of dental caries more than a century back.^1^ Even today, the modern concepts of cariogram demonstrate microorganisms as one of the major etiological factors apart from dietary factors and host factors in the formation of dental caries. It has been well established that mutans streptococci, particularly *Streptococcus mutans,* are an important caries-associated member of microorganisms in dental plaque.^2^ It induces mineral loss due to its adhesive and acidogenic potential resulting from the fermentation of carbohydrates, which keeps the local pH low.^3^ Hence, targeting *S. mutans* forms an important measure for the prevention of dental caries, which can be achieved by various mechanical and chemical aids. Many chemical bacteriostatic agents in the form of varnishes, dentifrices, and mouthwashes have been tried for improvement of oral health. Among the various mouthwashes available, the most persistent antimicrobial action has been achieved by chlorhexidine (CHX) mouthwash.^4^

Chlorhexidine is a powerful antimicrobial agent. One of its principal advantages is its property of substantivity. It has the ability of binding to a variety of substrates and at the same time maintains its antibacterial activity for a long period of time. At low concentrations, it is known to have a bacteriostatic action and at high concentrations it has a bactericidal property.^5^

Different preventive approaches have focused on the reduced sugar intake and its replacement with nonfer-mentable sweeteners, like polyols. Today, the most commonly used polyols are sorbitol and xylitol (XYL),^6^ which are incorporated in chewing gums. Unlike sorbitol, XYL has been observed to exhibit a dose-related inhibition of *S. mutans* growth *in vitro.*

Xylitol is a polyalcohol derivative that does not induce dental caries.^7,8^ Substitution of sugars by XYL is noncar-iogenic as well as anticariogenic,^9-12^ and it is mainly indicated for use as a sugar substitute between meals.^11^ As it is not metabolized by oral bacteria^8,13^ and leads to no pH drop in the biofilm,^14,15^ XYL also penetrates into the bacterial cytoplasm and accumulates as xylitol 5-phosphate, which impairs the glycolysis and adenosine triphosphate production and results in cell growth inhibition.^8,13^

Since both CHX mouthwash and XYL chewing gums have been found to reduce the levels of *S. mutans* when used individually by different modes of action, it can be postulated that their combination could produce a synergistic effect against *S. mutans* levels. Moreover, not many clinical trials have been conducted to test the efficacy of the combined use of both CHX mouthwash and XYL chewing gums against *S. mutans* levels. Hence, we conducted the present study to evaluate the effect of combining 0.2% CHX mouthwash (hexidine) with XYL chewing gum (extra XYL) in comparison with the individual agents of CHX mouthwash and XYL chewing gums on *S. mutans* and biofilm levels in 8- to 12-year-old children.

## MATERIALS AND METHODS

This study was undertaken in the Department of Pedodontics and Preventive Dentistry, ITS Dental College, Ghaziabad, India, in coordination with the Department of Microbiology, ITS Dental College, Ghaziabad, India. Prior to the study, an informed written consent was obtained from the parents. A randomized experimental study was designed and approved by the Ethical Committee, ITS Dental College, Ghaziabad, India. Initially, 500 children from a nearby school were examined, and 150 children were selected based on the following criteria.

### Inclusion Criteria

- Children in the age group of 8 to 12 years
- Caries-free children
- Children who agreed to participate in the study with the consent of parents.

### Exclusion Criteria

- Medically compromised children
- Children with a history of taking antibiotics 3 months prior to and during the study period
- Presence of any intraoral soft tissue pathology.

Baseline saliva samples were taken from these 150 patients and subjected to microbiological analysis. A sterile tongue blade (180 × 18 mm) was inserted into the child’s oral cavity and then moved around the buccal mucosa up to ten times, with both sides being then pressed on a Rodac^®^ plate (Kracjeler Scientific, Inc) containing 12 mL of mitis salivarius agar base (Becton, Dickinson & Company, Sparks, MD, USA) containing 0.2 g/mL sorbitol, 0.01 mg/mL potassium tellurite, 1.66 μg/mL bacitracin, and 1.275 μg/mL kanamycin sulfate.^16^

The plates were then incubated at 37°C for 72 hours in an anaerobic jar (BBL Gas Pak, Becton Dickinson and Co., Cockeysville, MD, USA) with an atmosphere of 80% N_2_, 10% H_2_, and 10% CO_2_. The period of time elapsed between inoculation and anaerobic incubation did not exceed 4 hours. Colony-forming unit (CFU) scores were counted in the spatula impression using a stereoscopic microscope. The CFU scores for *S. mutans* were expressed according to the criteria described by Weber as follows: 0 = absence of *S. mutans,* 1 = low level (1-10 CFU), 2 = moderate level (11-100 CFU), 3 = high level (101-250 CFU), 4 = very high level (>250 CFU).

A total of 60 children with CFU scores equal to or above moderate CFU level were included for further evaluation of the effect of antimicrobial agents. These children were also examined in order to investigate the amount of visible biofilm on the tooth surface. This procedure followed the criteria established by Ribeiro and Souza,^17^ as can be seen in Table 1. The biofilm scoring was done based on the criteria given in the table using a sterile gauze piece.

The children were randomly divided into three groups. In group I (XYL) (n = 20), XYL chewing gum was chewed by children twice a day, half an hour after breakfast and half an hour after dinner: In group II (CHX) (n = 20), 10 mL of CHX mouthwash at 0.2% concentration was used twice a day in the interval of 12 hours once after breakfast and once after dinner. In group III (XYL+CHX) (n = 20), XYL chewing gums were used by children once a day after breakfast, and CHX mouthwash was used once after dinner.

**Table 1:** **Table 1:** Criteria for evaluating the biofilm level according to Ribeirio and Souza^17^

*Scores*		*Description*	
0		Absence of biofilm	
1		Thin biofilm on anterior teeth only	
2		Thin, diffuse, easily removable biofilm on anterior and/or posterior teeth	
3		Thick biofilm adhered to anterior/posterior teeth only	
4		Thick biofilm firmly adhered to anterior teeth and thin biofilm on posterior teeth, or thick biofilm firmly adhered to posterior teeth and thin biofilm on anterior teeth	
5		Thick biofilm firmly adhered to posterior and anterior teeth	

For the CHX rinse, the children received one bottle of 0.2% CHX and were trained to rinse with 10 mL for 60 seconds in an undiluted form for 1 month and later in 1:1 dilution with water after 1 month. After completing the rinsing, the subjects were asked to expectorate the mouth rinse and not to eat or drink anything for half an hour after the rinse. A written instruction on how to use the mouth rinse was also given to the parents.

For using XYL chewing gums, the subjects were instructed to chew the gum for 5 minutes after meals.

Fresh saliva samples were then analyzed after 1, 3, and 6 months. Also the biofilm levels were evaluated at each interval. The microbiologist was blinded as to the grouping of the samples.

Data were compiled and analyzed by using the statistical program Statistical Package for the Social Sciences (SPSS) version 11.0. Analysis of variance (ANOVA) was with *post hoc* Bonferroni test for multiple comparisons at 5% significance level.

## RESULTS

The total sample size was 60 children (males 35, females 25) in the age range of 8 to 12 years with the mean age of 7.40 ± 0.669 years.

### Colony-Forming Units

**Graph 1:**
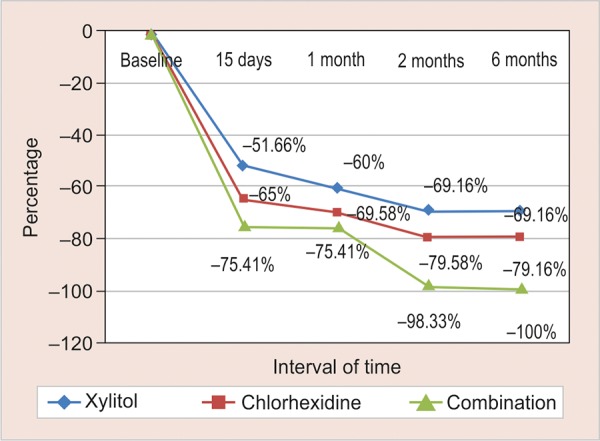
Percentage reduction in S. *mutans* scores using three different treatments at various time intervals

Table 2 shows that the mean baseline scores for CFU were 3.27 (group I), 3.27 (group II), and 3.06 (group III). In all the groups, significant reduction in *S. mutans* was found as compared with baseline values at four different time intervals (Table 3). As is evident from Graph 1, maximum reduction was seen at the end of 15 days, after that there was a gradual decline up to 2 months and later the difference was found to be insignificant.

At all the time intervals, there was significant difference of CFU between all the groups, with the maximum reduction seen in group III (100%) followed by group II (79%), and least reduction was seen in group I (69%).

On intergroup comparison of CFU by *post hoc* Bonferroni test, as is evident from Table 4, after 15 days, there was significant difference seen between groups I and II (p < 0.01) as well as between groups I and III (p < 0.001). There was no significant difference in the reduction observed between groups II and III (p > 0.05). After

1 month, there was significant difference seen between groups I and III (p < 0.01), but there was no significant difference observed between groups I and II (p > 0.05) as well as between groups II and III (p > 0.005). After

2 months, there was significant difference between all the groups (p < 0.001). After 6 months, there was significant difference seen between all the groups (p < 0.001).

**Table 2:** **Table 2:** Levels of biofilm and S. *mutans* in the different groups during the four time intervals

*Groups*		*Time* *interval*		*S. mutans* *CFU (mean* *scores)^a^*		*Biofilm* *(mean* *scores)^b^*	
I (XYL)		Baseline		3.27		3.40	
		15 days		1		0.47	
		1st month		0.73		0.27	
		2nd month		0.46		0.4	
		6th month		0.6		0.27	
II (CHX)		Baseline		3.27		3.6	
		15 days		0.73		1.13	
		1st month		0.87		0.33	
		2nd month		0.53		0.67	
		6th month		0.33		0.27	
III (XYL + CHX)		Baseline		3.06		3.00	
		15 days		0.67		0.67	
		1st month		0.13		0.6	
		2nd month		0.6		0	
		6th month		0		0	

^a^According to criteria by Weber

^b^According to criteria by Ribeiro and Souza

**Table 3:** **Table 3:** Intergroup comparision of S. *mutans* scores

*Time interval*		*Groups*		*p-value*	
15 days		I *vs* II		0.007**	
		II *vs* III		0.057 NS	
		I *vs* III		0.000***	
1 month		I *vs* II		0.082 NS	
		II *vs* III		0.519 NS	
		I *vs* III		0.002**	
2 months		I *vs* II		0.007**	
		II *vs* III		0.000***	
		I *vs* III		0.000***	
6 months		I *vs* II		0.004**	
		II *vs* III		0.000***	
		I *vs* III		0.000***	

*Statistically significant (p < 0.05); **Highly significant (p < 0.01);

***Very highly significant (p < 0.001); NS: Nonsignificant (p > 0.05)

**Graph 2:**
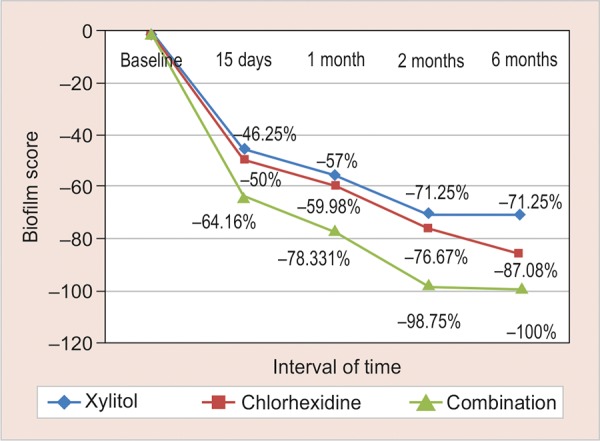
Percentage reduction in biofilm scores using three different treatments at various time intervals

**Table 4:** **Table 4:** Intergroup comparison of biofilm scores

*Time interval*		*Groups*		*p-value*	
		I *vs* II		1.00 NS	
15 days		II *vs* III		0.028*	
		I *vs* III		0.004**	
		I *vs* II		1.00 NS	
1 month		II *vs* III		0.009**	
		I *vs* III		0.002**	
		I *vs* I		0.199 NS	
2 months		II *vs* III		0.000***	
		I *vs* III		0.000***	
		I *vs* II		0.000***	
6 months		II *vs* III		0.000***	
		I *vs* III		0.000***	

*Statistically significant (p < 0.05); **Highly significant (p < 0.01);

***Very highly significant (p < 0.001); NS: Nonsignificant (p > 0.05)

### Biofilm Scores

Table 2 shows the mean baseline scores for biofilm as 3.40 (group I), 3.6 (group II), and 3.00 (group III). In all the groups, significant reduction was seen at different time intervals. As is evident from Graph 2, maximum reduction of biofilm was seen at the end of 15 days; after that there was a gradual decline from 2 to 6 months in groups I and II, whereas there was a sharp decline in group III during this period.

At all the time intervals, there was significant difference in the reduction of biofilm scores between all the groups, with the maximum reduction seen in group III. Groups I and II showed similar difference in reduction at 15 days, 1 month, and 2 months (p > 0.05), but group II showed better reduction than group I at 6 months (p < 0.001). Group III showed significant reduction at 15 days (64%), 1 month (78%), 2 months (98%), and 6 months (100%).

## DISCUSSION

The pellicle, which is an organic bacteria-free film, deposits on the tooth surface after toothbrushing or polishing and leads to the beginning of biofilm formation. During this time, *S. mutans* becomes an important factor in the modification of the biofilm into a cariogenic form.^18^ As the level of *S. mutans* increases, the level of plaque accumulation also increases, and this leads to greater risk of developing dental caries. Hence, the control of *S. mutans* levels is one of the important targets for caries prevention and control.

In this study, the spatula method was used for saliva collection as it was more practical for children unlike other classical methods in which saliva is collected after stimulation. Moreover, the saliva collected does not need to be diluted before inoculation, making it more appropriate for epidemiological studies.

The mitis salivarius, sorbitol, kanamycin, and bacitra-cin agar medium was used as it is more selective for the *S. mutans* colonies with a long shelf life, and there are a reduced number of nonmutans colonies that could have been confused visually with *S. mutans.^17^*

As shown by long-term clinical trials, the use of antimicrobial agents in the oral cavity may reduce the salivary *S. mutans* levels. Therefore, the use of chemotherapeutic regimens is being advocated with a potential of chemical control of dental biofilm and consequent caries prevention.^19^ Out of all the several chemical agents used, CHX and XYL have been observed to have strong antimicrobial activity with different modes of action.

Chlorhexidine is considered to be the gold standard among all the chemical agents used due to its prolonged and broad spectrum antimicrobial activity. At high concentrations, it is known to act as a bactericidal agent. At low concentrations, it has a bacteriostatic effect.^5^ In the present study, the children who used 0.2% CHX mouthwash showed significant reduction in the biofilm levels at the end of 6 months (p < 0.001). Complete inhibition of bacterial accumulation has been reported by Schiott et al.^20^ Jarvinen et al^21^ showed that CHX was highly effective against all the *S. mutans* isolates in which the minimum inhibitory concentration did not exceed 1 ug/mL for any of the isolates. In the analysis performed at the end of our study (6th month), biofilm reductions were 87% for CHX group. These results collaborate well with the results obtained in a previous study done by Clark and Guest,^22^ who found greater reduction in the number of *S. mutans* in subjects who used 0.12% CHX mouthwash as compared with XYL chewing group. In our study, the reduction in the number of *S. mutans* was found to be statistically significant at the end of 6 months, i.e., 100% (p = 0.000). Wan et al^23^ in a study done on infants using CHX gel achieved reduction of *S. mutans* to 0 CFU/mL in 41% of the children. Kulkarni and Damle compared the efficacy of sodium fluoride (0.05%), CHX mouthwash (0.12%), and triclosan (0.3%) mouth rinses in the reduction of mutans streptococci count in saliva. They found that CHX mouth rinse showed absolute reduction of 2.280 × 10^5^ CFU/mL of saliva, when compared with sodium fluoride (1.400 × 10^5^ CFU/mL) and triclosan mouth rinse (1.460 × 10^5^ CFU/mL).^24^

In recent times, various polyalcohols have been incorporated into products like chewing gums and mouth rinses. Xylitol inhibits the glycolysis pathway resulting in the formation of loosely attached biofilms. However, the use of XYL as a sugar substitute in food does not result in decreased salivary *S. mutans* levels.^25,26^ Therefore, the frequent and sustained effect of XYL in the form of candies or gums is required in order to achieve reduced *S. mutans* levels.^26,27^

Few studies have demonstrated a XYL-associated decrease of MS counts in plaque (Makinen et al; Milgrom et al; Haresaku et al)^28,29^ and in resting saliva (Milgrom et al) and stimulated saliva (Haresaku et al).^30^ In a 2-year study, the *S. mutans* levels in plaque of 11- to 12-year-old children decreased and remained low throughout the study (Makinen et al).^31^ In another 2-year study, however, no significant decrease could be observed in the salivary *S. mutans* counts of 10-year-old children (Makinen et al).^28^ In the XYL group, initial *S. mutans* suppression was observed after 15 days when compared with baseline. These results were statistically significant, which agree with other studies in which the *S. mutans* CFU reduction did not persist for a long time after XYL therapy. Several studies have also demonstrated the short-term effect of polyol on *S. mutans,* and in our study, we observed the CFU scores to be consistent from 2 to 6 months in group I (69.16%). In another study, Moraes^33^ showed that at the end of 3 months, the XYL group returned to mean baseline scores (2.67) and remained the same at 6 months.^33^ Similarly, Hildebrandt et al^34^ demonstrated that 4.4 gm/day of XYL mouth rinsing did not show a significant decrease of MS level, although a 1 log unit reduction was observed, whereas Arunakul et al^35^ revealed a significant reduction of MS scores following chewing XYL gum at a dose of 5.8 gm/day for 3 months.

It has been suggested that because of the different mechanisms of action of CHX and XYL, using a combination of these antimicrobial agents can give better results rather than the individual agents used alone. Moreover, CHX is reported to have side effects like transient change in oral flora, altered taste sensation, and brown staining of teeth if used for long period of time,^36^ and XYL at high dosage can cause gas and osmotic diarrhea.^37^ Also, long-term XYL use has shown the development of XYL-resistant strains.^38-40^ Thus, it can be assumed that such pairing can reduce adverse effects as their combination will reduce the frequency of application. Hence, in our study, we have used both CHX (mouthwash) and XYL (chewing gums) and assessed its antimicrobial efficiency in comparison to the use of individual agents.

The results of our study showed enhanced outcomes from this combination as is evident by the maximum reduction of *S. mutans* CFUs (100%) and biofilm levels (100%) in group III at the end of 15 days, 1, 2, and 6 months. In 2010, Paula et al^41^ showed that the lowest biofilm levels were seen in the combination of CHX varnish and XYL group at the end of 1st month (40%), 2nd month (29%), and 6th month (46%), with statistical significance for the three time intervals (p < 0.05). With regard to *S. mutans* levels at the end of 6 months, the largest reduction (100%) was also observed in group III (CHX + XYL), whereas group II (CHX) showed 79.16% reduction. In an *in vitro* study, it was found that the combination of CHX + XYL performed a synergistic action, and multiple exposures of *S. mutans* to CHX followed by secondary exposures to XYL exhibited a transient inhibition of growth within 24 hours and significantly decreased the ability of *S. mutans* to form biofilms.^42^

Chlorhexidine mouthwash and XYL chewing gums were used for the present study as they have the advantage of being economical and applicable at the community level and do not require any clinical setup. Subjects between 8 and 12 years were the target of this research as it is the late mixed dentition stage, and the combination of CHX mouthwash and XYL chewing gums for children has not been previously investigated.

In our study, no stains or irritated gingival tissue was observed on the teeth at any of the follow-up period in either of the CHX group or CHX+XYL group. Diarrhea was also not reported in any of the children in the XYL group.

Even in a short period of time, which is the limitation of this study, these agents proved to be effective as observed in the results achieved. Although the results of our study are promising, a longer follow-up evaluation is required for this type of preventive procedure in high-risk caries children who are at the initial stage of caries development.

## CONCLUSION

Based on this study’s results, the following conclusions can be made:

- Overall reduction in *S. mutans* counts was observed in all the treatment groups, with the combination of CHX+XYL presenting the maximum reduction.
- All the groups also presented a reduction in the biofilm levels with combination of CHX+XYL showing the maximum reduction.
